# Ellagic acid attenuates beryllium sulphate-induced oxidative stress and histopathological alterations of spleen in rats

**DOI:** 10.1080/13880209.2022.2074051

**Published:** 2022-06-01

**Authors:** Yuandi Lei, Tianyi Jiang, Liqin He, Yanping Liu, Zhanbing Sun, Weihua Deng, Lian Huang, Zhaohui Zhang

**Affiliations:** Department of Preventive Medicine, School of Public Health, Hengyang Medical School, University of South China, Hengyang, Hunan, China

**Keywords:** Beryllium sulphate, ellagic acid, spleen, oxidative stress, apoptosis

## Abstract

**Context:**

Ellagic acid (EA) is a phenolic constituent in certain fruits and has largely been recognized for its role as an antioxidant compound.

**Objective:**

To evaluate the effect of EA on beryllium sulphate-induced splenic toxicity in rats.

**Materials and methods:**

Male Sprague-Dawley rats were divided into four groups. The first group was used as control. Group 2 was exposed to BeSO_4_ (12 mg/kg, b.w.). Groups 3 and 4 were treated with EA (100 and 300 mg/kg, b.w.) daily for 6 weeks after exposing to BeSO_4_ (12 mg/kg, b.w.). Various biochemical and molecular biomarkers were assessed in blood and spleen.

**Results:**

BeSO_4_-intoxicated rats showed significant higher WBC (6.74 ± 0.20 × 10^9^/L vs. 11.02 ± 1.31 × 10^9^/L, *p* < 0.05), Neu (1.14 ± 0.11 × 10^9^/L vs. 2.45 ± 0.42 × 10^9^/L, *p* < 0.05), Lym (3.80 ± 0.83 × 10^9^/L vs. 9.64 ± 1.99 × 10^9^/L, *p* < 0.05), and PLT (868.4 ± 43.2 × 10^9^/L vs. 1408 ± 77.57 × 10^9^/L, *p* < 0.05) than normal control animals. Moreover, an increase in MDA with depletion of GSH and SOD activity (all *p* < 0.05) occurred in the spleen of rats treated with BeSO_4_. Furthermore, BeSO_4_-treated rats displayed significantly higher levels of apoptotic markers (Bax, Caspase-3, PARP) (all *p* < 0.05). EA administration resulted in a significant reversal of hematological and apoptotic markers in beryllium sulphate-intoxicated rats.

**Discussion and conclusions:**

Our results suggest EA treatment exerts a significant protective effect on BeSO_4_-induced splenic toxicity in rats.

## Introduction

Beryllium (Be) and its compounds are highly desirable due to its excellent properties such as enhancing metal-hardening capacity, high electrical and thermal conductivity, high melting and boiling points (Kreiss et al. 2007). They are widely used in the industrial fields, including aircraft, space shuttle brakes, satellite mirrors, electrical circuits, computer components, nuclear weapons, home appliances, etc. Unfortunately, Be is highly toxic. It has been demonstrated that the LD_50_ value of beryllium nitrate is 3.16 mg/kg (Mathur et al. 1985). Exposure to Be and its compounds could induce a wide range of diseases such as acute chemical pneumonia (Cummings et al. 2009), chronic beryllium disease (Ribeiro et al. 2011), even lung cancer (Levy et al. 2009). Be exposure occurs primarily through the inhalation of Be particulates that induces oxidative stress and leads to various pathological consequences (Sawyer et al. 2005). Thus, inhibition of oxidative stress may be an approach in the prevention of beryllium-related diseases.

Spleen is the largest organ of the lymphatic system, which plays an important role in immune functions such as recycling old red blood cells and serving as a repository for platelets and white blood cells (Mebius and Kraal 2005). Unfortunately, the spleen is sensitive to chemical pollutants. Based on a series of convincing reports regarding spleen susceptible properties, it was considered that Be and its components might also induce toxicity in the spleen. However, the studies on Be related diseases mainly focussed on the lung and no research into the spleen in Be exposure has been done.

Numerous synthetic compounds have been used to against Be intoxication but they have certain limitations. Increasing interest towards natural products for the treatment of a variety of diseases attracted us to deal beryllium toxicity with some plant extracts having phenolic constituents. Ellagic acid (2,3,7,8-tetrahydroxy-benzopyrano 5,4,3-cdebenzopyrano-5,12-dione, EA) is a phenolic constituent found in various plants such as raspberry, strawberry, walnut, and pomegranate (Soong and Barlow 2006). EA has largely been recognized for its role as an antioxidant (García-Niño and Zazueta 2015). The potent scavenging action against ROS of EA such as hydroxyl and superoxide radicals are well documented (Priyadarsini et al. 2002). Also, EA exerts its antioxidant effect via activating antioxidant defense systems such as SOD, GSH, and CAT (Goudarzi et al. 2018). Previous studies have demonstrated that EA has protective effect on oxidative damage in the lung (Gul et al. 2013) and liver (Yüce et al. 2007). In addition, EA is a safe substance, Doyle and Griffiths (1980) reported that feeding rats with EA (50 mg/day up to 45 days) does not cause any sign of systemic toxicity.

In the present study, we investigated the protective effect of EA against BeSO_4_-induced splenic toxicity in SD rats with a focus on oxidative stress.

## Materials and methods

### Chemicals

Beryllium sulphate tetrahydrate (BeSO_4_·4H_2_O) was purchased from Sigma-Aldrich (St. Louis, MO, USA). Ellagic acid (EA) was obtained from McLean Biochemical Technology Co., Ltd. (Shanghai, China). All other chemicals and reagents used were bought from Servicebio Technology Co., Ltd. (Wuhan, China).

### Maintenance of animals and their feeding

Forty specific-pathogen-free (SPF) male Sprague Dawley (SD) rats (six-week-old, weight 174 ± 9.0 g) were purchased from animal house of Hunan Changsha Tian Qin Biological Experimental Animals Limited Liability Company. Rats were raised at stranded conditions (20‐25 °C, relative 45–55% humidity, 12 h light/dark cycle) with free access to water and food for 14 days. All animals for the studies were handled, anaesthetized, and sacrificed in accordance with the China Animal Protection Law and the protocols were approved by the University of South China of Technology Animal Care and Use Committee (Approval number:SYXK2019-0004).

### Preparation of doses and treatments

Beryllium sulphate tetrahydrate (4.05 g) (99% w/v; Sigma-Aldrich, St. Louis, MO, USA) was diluted in 200 mL distilled water making up doses of 12 mg/mL BeSO_4_ solution and administered intratracheally (Duckett et al. 2000). The doses of ellagic acid (100 and 300 mg/10 mL) were prepared in 1% carboxymethyl cellulose and administered by gavage (El-Shitany et al. 2014). Forty rats were acclimated for 2 weeks prior to the experiment and randomly separated into four groups, with 10 rats in each group.

Group 1: Labelled as control and administered with distilled water.
Group 2: BeSO_4_ (12 mg/kg, b.w.).
Group 3: BeSO_4_ (12 mg/kg, b.w.) + EA (100 mg/kg, b.w. daily for 6 weeks).
Group 4: BeSO_4_ (12 mg/kg, b.w.) + EA (300 mg/kg, b.w. daily for 6 weeks).

All rats were administered daily at 6 pm. Water and food was available *ad libitum* for the duration of the study. Body weights were measured every day.

The dose of reagents has been considered according to the previous studies (Duckett et al. 2000; El-Shitany et al. 2014).

### Sample collection

After the final administration, rats were sacrificed under mild anaesthesia by using ether. Blood samples were collected through abdominal aorta and stored at −20 °C until analysis. Spleens were quickly isolated, washed, weighted, and immediately frozen in liquid nitrogen and stored at −80 °C for further evaluation. Standard techniques were applied to assay various biochemical parameters.

### Hematological analyses

The whole blood cell count was used for hematological analysis. The following hematological parameters including the white blood cell (WBC), neutrophil (Neu), lymphocyte (Lym) and platelet (PLT) counts were analysed immediately by a haematology system (Sysmex XE-2100, Japan) according to the manufacturer’s instructions.

### Quantification of total cell population in the spleen

Total splenocytes were isolated from sacrificed animals and placed in phosphate buffering saline (PBS) at concentrations of 1 mg/mL. Spleens were gently homogenized with a piston syringe in PBS and then passed through a 200-mesh cell strainer. Afterwards, the supernatants were removed and the pellets were treated with an equal volume of red blood cell lysis buffer (150 mM NH_4_Cl, 1 mM KHCO_3_, and 110 mM Na_2_EDTA, pH 7.2) for 1 min at 4 °C. Cell suspensions were then centrifuged at 1000 rpm for 5 min. After washing twice with PBS, the pellets were suspended in RPMI-1640 supplemented with 10% foetal calf serum, 100 U/mL penicillin, and 100 μg/mL streptomycin. Finally, the number of cells was counted using haemocytometers.

### Proliferation assay with primary cultured splenocytes

Briefly, splenocytes were suspended in RPMI-1640 complete medium containing 12% FBS, 100 U/mL penicillin and 100 µg/mL streptomycin, seeded into 96-well plates at 5 × 10^4^ cells/well and incubated with or without either Con A (5 μg/mL) or LPS (5 μg/mL) at 37 °C for 48 h in a humidified air with 5% CO_2_. Afterwards, 50 μL MTT reagent (5 mg/mL) was added to each well, incubation was continued for 6 h. Finally, 100 μL DMSO was added to each well and left to stand for 15 min, and the absorbance value was measured at 570 nm using a microplate reader (Bio-TEK, USA).

### The determination of reduced glutathione (GSH) content, superoxide dismutase (SOD) activity and malondialdehyde (MDA) levels in the spleen of BeSO_4_-intoxant rats

GSH content was measured fluorometrically using orthophthalaldehyde (OPT) according to the method described previously (Hissin and Hilf 1976). The activity of SOD was measured according to Kakkar et al. (1984) using commercial ELISA kits (Jiancheng Bioengineering Institute, Nanjing, China). The splenic lipid peroxidation was determined by using MDA assay kit (Servicebio, China), which estimates the malondialdehyde (MDA) formation.

### RNA isolation, cDNA synthesis and quantitative real-time PCR

RNA was isolated from the spleen of rats of each group using Trizol reagent (Servicebio, China) as mentioned in the manufacturer instructions. Quantitative real-time PCR (qRT-PCR) was performed by using Light Cycler^®^ 480 II Real-time PCR Instrument (Roche, Swiss). Briefly, cDNA was synthesized with miScript Reverse Transcriptase Mix according to manufacturer's instructions. Reaction mixture containing 2 μL cDNA, 12 μL 2 × QuantiFast^®^ SYBR^®^ Green PCR Master Mix (Servicebio, China), 0.2 μL universal primer (Servicebio, China), 2 μL miRNA-specific primer and 6 μL nuclease-free water was incubated at 55 °C for 5 min. Each sample was run in triplicate for analysis. Reactions were incubated in a 384-well optical plate (Roche, Swiss). Amplification was done using specific primers (2.5 pmol/µL). Primer sequences used were GAPDH (forward 5′‐CTG GAG AAA CCT GCC AAG TAT G‐3′; reverse 5′‐GGT GGA AGA ATG GGA GTT GCT‐3′), Bax (forward 5′‐GGC GAT GAA CTG GAC AAC AAC‐3′; reverse 5′‐CCC AGT TGA AGT TGC CGT CT‐3′), Bcl-2 (forward 5′‐TTG TGG CCT TCT TTG AGT TCG‐3′; reverse 5′‐GCA TCC CAG CCT CCG TTA T‐3′), Caspase-3 (forward 5′‐GAA AGC CGA AAC TCT TCA TCA T‐3′; reverse 5′‐ATG CCA TAT CAT CGT CAG TTC C‐3′), PARP (forward 5′‐CAA GTC CAA CAG GAG CAC ATG‐3′; reverse 5′‐TTC CAT CCA CCT CGT CAC AT‐3′). PCR was programmed at 95 °C for 12 min, followed by 40 cycles of 90 °C for 15 s and 60 °C for 30 s. GAPDH was used as an internal control, and the relative expression of the target gene was calculated after amplification using the 2^-ΔΔCT^ method.

### Western blotting

Spleen tissues were homogenized in ice‐cold lysis buffer and centrifuged at 12,000 rpm for 10 min at 4 °C. The supernatant was collected for immunoblots. The proteins were electrophoresed under reducing conditions in 12% sodium dodecyl sulphate‐polyacrylamide gel electrophoresis (SDS-PAGE) and transferred to the polyvinylidene difluoride (PVDF) membrane. The membranes were then blocked with 5% non-fat dry milk followed by overnight incubation at 4 °C with corresponding antibodies (anti‐Bax, 1:1000; anti‐Bcl-2, 1:1000; anti‐caspase-3, 1:1000; anti‐PARP, 1:1000) and β-actin (1:2000). After washing with TBS-T (0.01% of Tween-20 in TBS), membranes were incubated with alkaline phosphatase conjugated anti-Rabbit IgG antibody at room temperature in the dark for 1 h and detected by the gel image analyser system (Bio-Rad, Universal HodII, USA).

### Histopathological spleen damage

For the histopathological examination, spleens of 5 randomly selected rats from each group were isolated and fixed in 4% paraformaldehyde PBS solution. Afterwards, tissues were dehydrated, imbedded in paraffin wax, and cut into 5 μm thick sections using microtome (Lecai, Germany). The sections were stained with haematoxylin and eosin (H&E) for microscopic examination.

### Statistical analysis

The results were expressed as mean ± SD. SPSS 20.0 statistical software was used to analyse the experimental data, one way analysis of variance (ANOVA) was used for the comparison between groups, and *p* < 0.05 was considered statistically significant.

## Results

### Beryllium sulphate-induced alterations in the spleen weight are attenuated by EA

**Figure 1.**
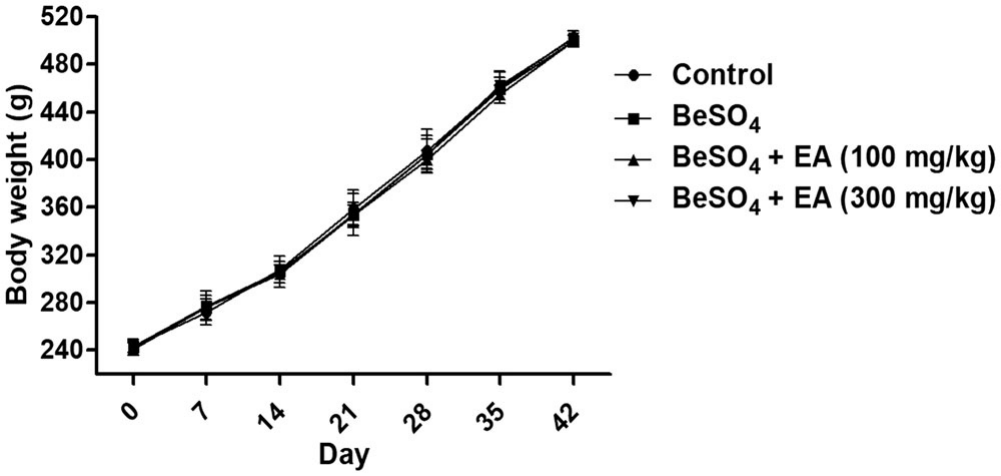
Change in the body weight (BW) during the experiment/Body weights were measured every day. Data are represented as mean ± SD (*n* = 10). Data were analysed by one way analysis of variance (ANOVA). *p < 0.05: Significant difference in comparison with the control group. ^#^*p* < 0.05: Significant difference in comparison with the BeSO_4_-treated group.

As shown in Figure 1, there were no significant differences between the control group, the BeSO_4_-treated group and EA treated groups in regard to the body weight of the rat for the duration of the study. When compared to the control group, treatment with 12 mg/kg BeSO_4_ caused 18% increase in the spleen weight and 19% increase in the relative spleen weight to the body weight, respectively (Table 1). Treatment with EA (100 and 300 mg/kg) caused 12 and 16% decreases in the spleen weight and 12 and 16% decreases in the relative spleen weight to the body weight respectively compared to the BeSO_4_-treated group (Table 1).

**Table 1.** Effect of EA on the spleen weight in BeSO_4_-intoxicant rats.

Groups	Final body weight (g)	Spleen weight (g)	Relative spleen weight to the body weight (%)
Control	502.81 ± 5.13	0.216 ± 0.003	0.042 ± 0.009
BeSO_4_	499.79 ± 3.90	0.255 ± 0.003*	0.051 ± 0.005*
EA 100	499.70 ± 4.77	0.225 ± 0.001^#^	0.045 ± 0.004^#^
EA 300	502.03 ± 3.40	0.215 ± 0.002^#^	0.043 ± 0.004^#^

After the final administration, animals were sacrificed by decapitation and spleen tissue samples were weighted and the weight of the spleens relative to the body weight was calculated.

Data are represented as mean ± SD (*n* = 10). Data were analysed by one way analysis of variance (ANOVA).

*p < 0.05: Significant difference in comparison with the control group.

^#^*p* < 0.05: Significant difference in comparison with the BeSO_4_-treated group.

### Beryllium sulphate-induced alterations in hematological parameters are attenuated by EA

As depicted in Table 2, compared to the control group, treatment with 12 mg/kg BeSO_4_ caused significant 0.6-, 1.1-, 1.5-, and 0.6-fold increases in the WBC, Neu, Lym, and PLT, whereas administration of EA (100 and 300 mg/kg) mitigated the effect of BeSO_4_ on the above-mentioned variables (all *p* < 0.05), reflecting the amelioration of hematotoxicity in BeSO_4_-intoxicated rats. When compared to the BeSO_4_-treated group, treatment with EA at the dose of 100 mg/kg caused significant 0.2-, 0.3-, 0.3- and 0.2-fold decreases in the WBC, Neu, Lym, and PLT. Moreover, treatment with EA at the dose of 300 mg/kg caused significant 0.4-, 0.6-, 0.6- and 0.4-fold decreases in the WBC, Neu, Lym, and PLT compared to that of the BeSO_4_-treated group.

**Table 2.** Effect of EA on hematological parameters in BeSO_4_-intoxicant rats.

	Control	BeSO_4_	EA 100	EA 300
WBC (10^9^/L)	6.74 ± 0.20	11.02 ± 1.31*	9.22 ± 0.63^#^	7.03 ± 0.82^#^
Neu (10^9^/L)	1.14 ± 0.11	2.45 ± 0.42*	1.66 ± 0.13^#^	1.08 ± 0.25^#^
Lym (10^9^/L)	3.80 ± 0.83	9.64 ± 1.99*	6.37 ± 1.28^#^	3.92 ± 0.96^#^
PLT (10^9^/L)	868.4 ± 43.2	1408 ± 77.57*	1194 ± 28.90^#^	872.4 ± 86.4^#^

Blood sample was collected from the abdominal aorta. The whole blood cell count was used for hematological analysis.

WBC: white blood cell count; Neu: neutrophil count; Lym: lymphocyte count; PLT: platelet count.

Data are represented as mean ± SD (*n* = 5). Data were analysed by one way analysis of variance (ANOVA).

*p < 0.05: Significant difference in comparison with the control group.

^#^*p* < 0.05: Significant difference in comparison with the BeSO_4_-treated group.

### Beryllium sulphate-induced alterations in splenocyte proliferation are attenuated by EA

**Figure 2.**
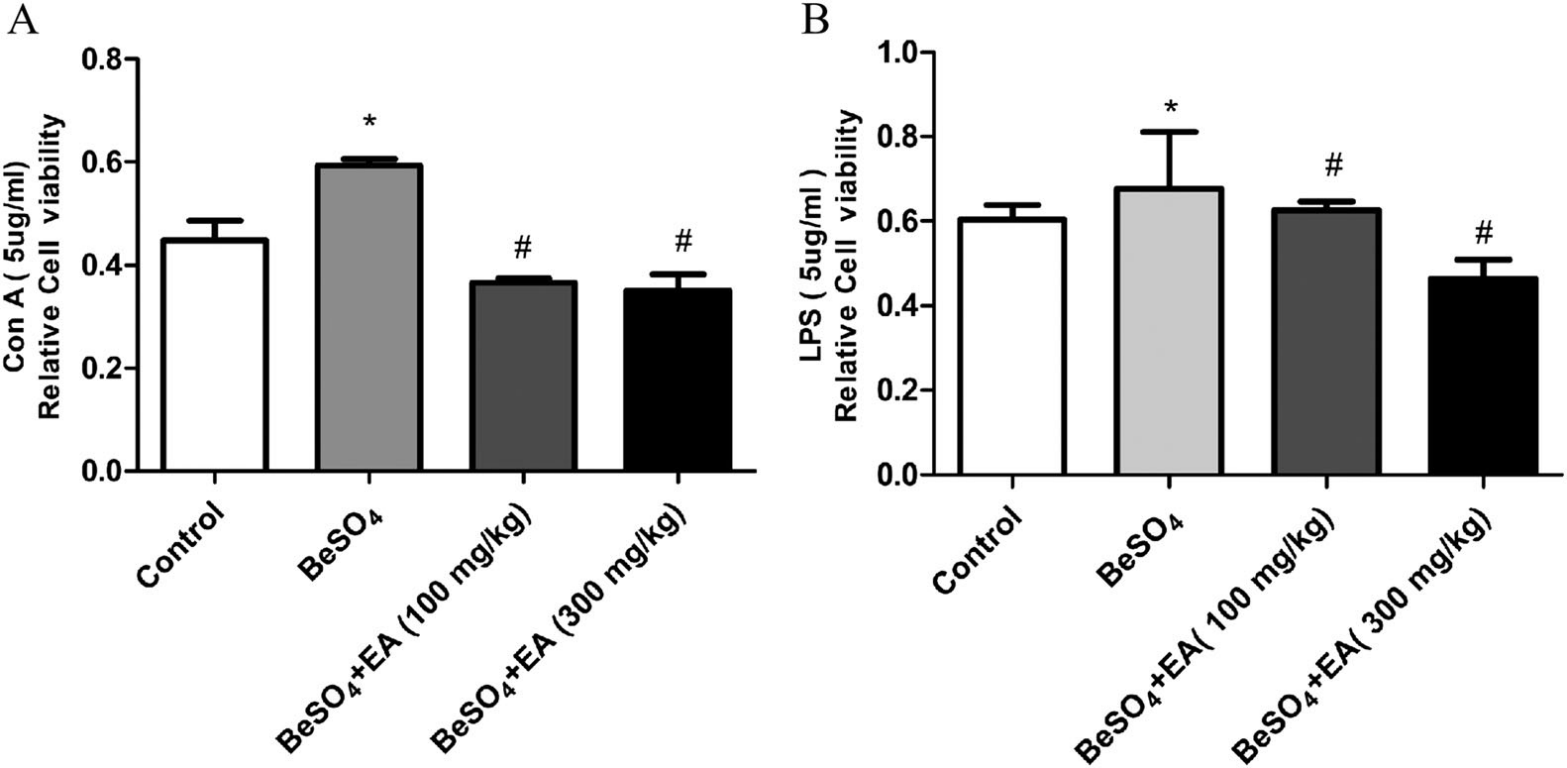
Effect of EA on splenocyte proliferation in BeSO_4_-intoxicant rats/Cultured splenocytes were suspended in RPMI-1640 complete medium containing 12% FBS, 100 U/mL penicillin and 100 µg/mL streptomycin, seeded into 96-well plates at 5 × 10_4_ cells/well and incubated with or without Con A (A) or LPS (B). Data are represented as mean ± SD (*n* = 5). Data were analysed by one way analysis of variance (ANOVA). *p < 0.05: Significant difference in comparison with the control group. ^#^*p* < 0.05: Significant difference in comparison with the BeSO_4_-treated group.

As shown in Figure 2, compared to the control group, treatment with 12 mg/kg BeSO_4_ caused 40 and 16% increases in the ConA and LPS-stimulated splenocyte proliferation. EA (100 and 300 mg/kg) treatment resulted in 34 and 36% decreases in the ConA-stimulated splenocyte proliferation and 21 and 26% decreases in the LPS-stimulated splenocyte proliferation, respectively, compared to the BeSO_4_-intoxicant rats.

### Beryllium sulphate-induced alterations in antioxidant enzymes are attenuated by EA

**Figure 3.**
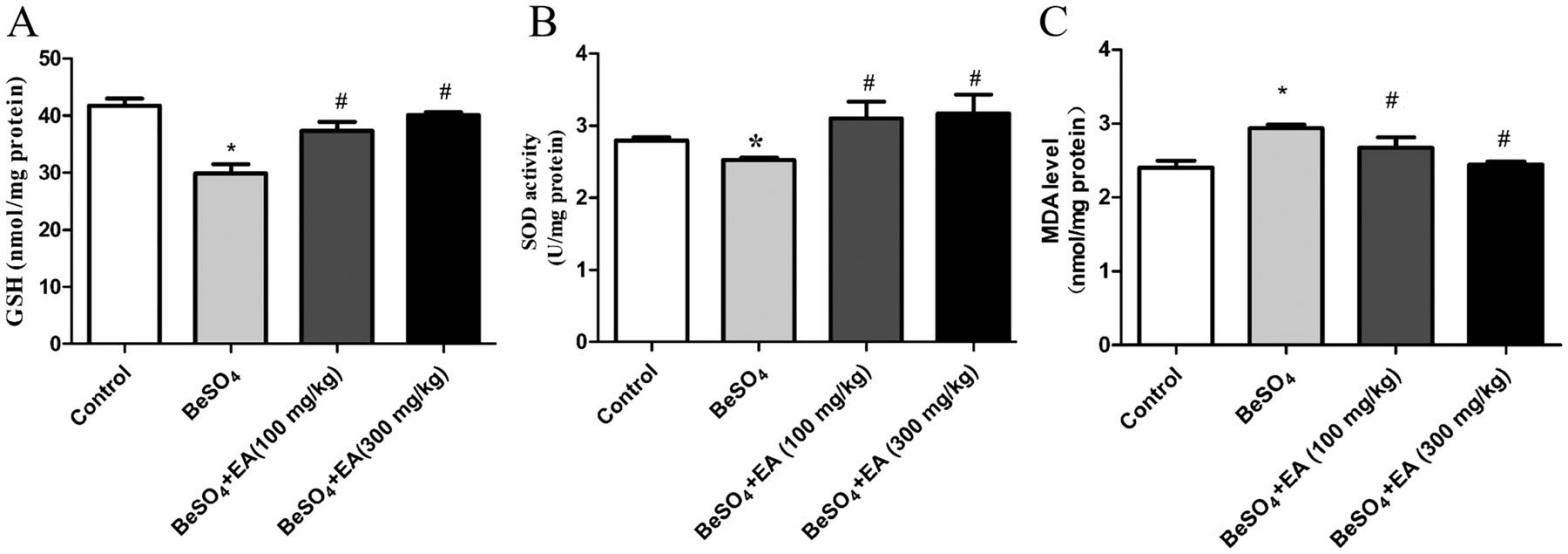
Effect of EA on GSH content, SOD activity and MDA level in BeSO_4_-intoxicant rats/GSH content was measured fluorometrically using orthophthalaldehyde (A). The SOD activity was measured using commercial ELISA kits (B). The level of MDA was measured using MDA assay kit (C). Data are represented as mean ± SD (*n* = 5). Data were analysed by one way analysis of variance (ANOVA). *p < 0.05: Significant difference in comparison with the control group. ^#^*p* < 0.05: Significant difference in comparison with the BeSO_4_-treated group.

EA was found to be effective in maintaining the content of GSH, activity of SOD and depleting the MDA level in the spleen. As shown in Figure 3, compared to the control group, the GSH (*p* < 0.05) content and the SOD (*p* < 0.05) activity were significantly decreased and the level of MDA (*p* < 0.05) was significantly increased after exposure to BeSO_4_. These effects were alleviated to normal levels due to EA treatment, in a dose-dependent fashion. Upon the EA (100 and 300 mg/kg) administration, the contents of GSH were increased 25 and 34%, the activities of SOD were increased 23 and 25%, the levels of MDA were decreased 9 and 17%, respectively compared to the BeSO_4_-intoxicant rats.

### Beryllium sulphate-induced alterations in apoptotic markers of the spleen are attenuated by EA

**Figure 4.**
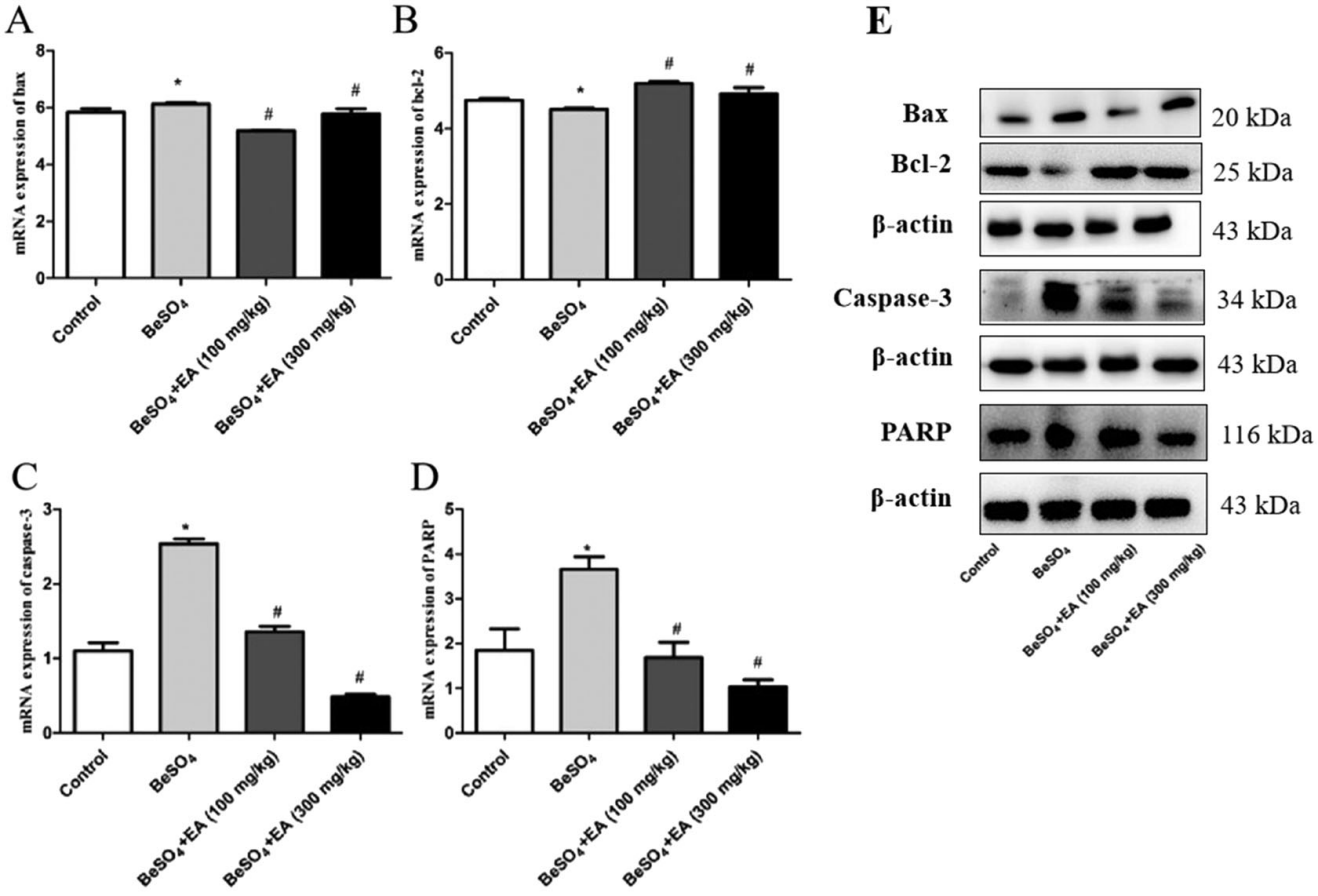
Effects of EA on apoptosis in BeSO_4_-intoxicant rats/Relative mRNA expression levels of Bax, Bcl-2, Caspase-3, and PARP are shown in Figure 3 (A,B,C,D). The expression levels of Bax, Bcl-2, Caspase-3, and PARP proteins were detected by western blotting are shown in Figure 4 E. Data are represented as mean ± SD (*n* = 5). Data were analysed by one way analysis of variance (ANOVA). *p < 0.05: Significant difference in comparison with the control group. ^#^*p* < 0.05: Significant difference in comparison with the BeSO_4_-treated group.

The expression of mRNA and proteins involved in apoptosis was assessed through qRT-PCR and Western blot using the spleen samples. As shown in Figure 4, the expression of Bax was up-regulated and the expression of Bcl-2 was downregulated in the BeSO_4_-treated group than the control group (*p* < 0.05). A dose-dependent increase in Bax and decrease in Bcl-2 were observed upon EA treatment. Further experiments were conducted to examine the involvement of caspase-3 and PARP. The activation of caspase-3 and PARP in the spleen samples from the BeSO_4_-treated group suggested apoptotic behaviour due to splenic toxicity. EA treatment showed inhibition of caspase-3 activation along with inhibited activation of PARP, which reveals the protective behaviour of EA against BeSO_4_ induced apoptosis. The expression of housekeeping gene β-actin was constant in all the experimental protein samples.

### Beryllium sulphate-induced alterations in histopathology of the spleen are attenuated by EA

**Figure 5.**
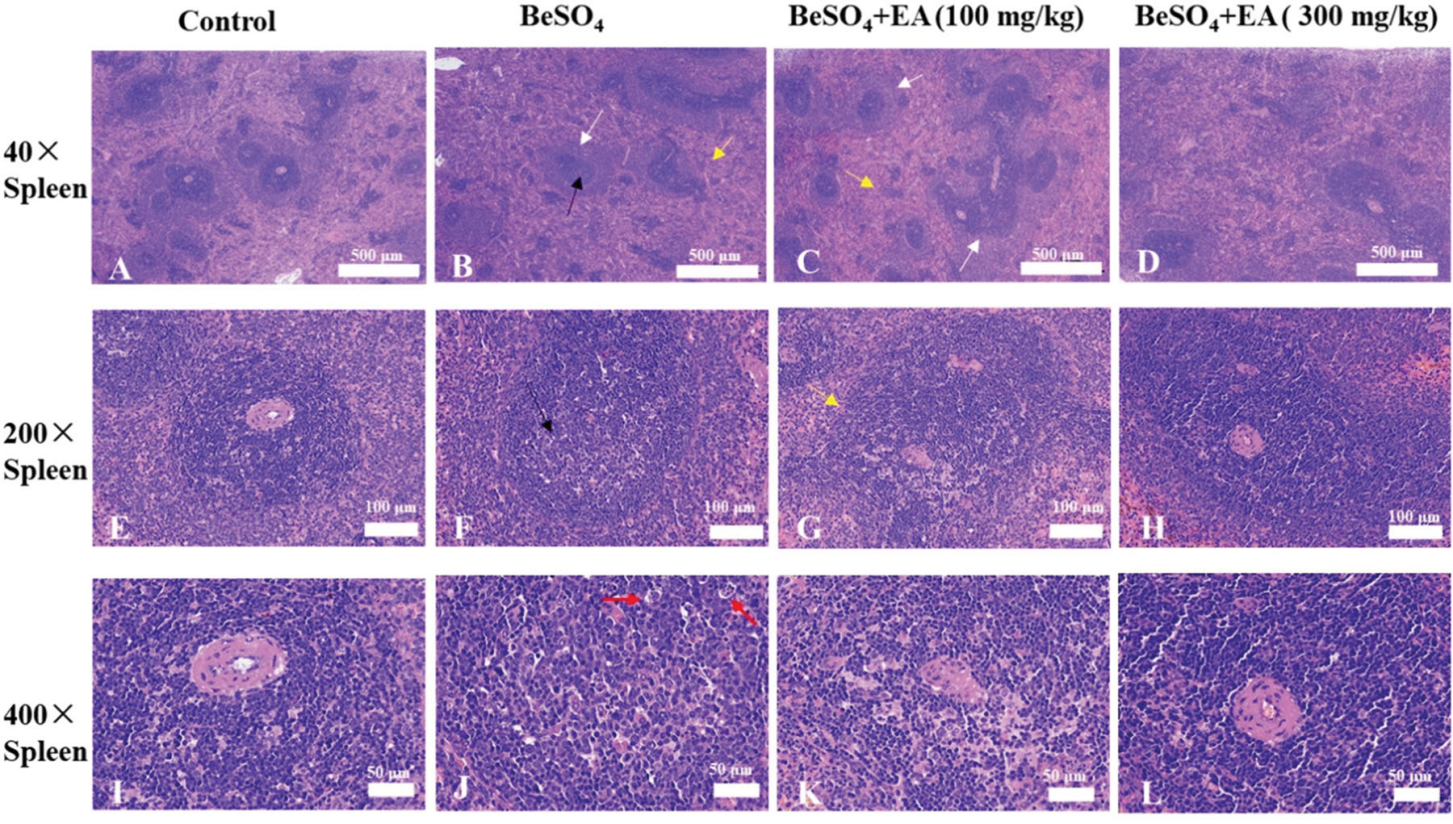
Histopathological observations showing effects of EA on BeSO_4_-induced changes in spleen/Spleen tissues were subjected to H&E staining. Microscopic images were taken at a magnification of 40 × (A–D), 200 × (E–H) and 400 × (I–L) for the spleen. White arrow: thickening of the marginal area; Black arrow: localized central dilatation of the spleen nodules; Yellow arrow: red pulp with blood stasis; Red arrow: nuclear fragmentation.

The results of microscopic studies in the spleen tissues of different experimental groups are shown in Figure 5. Compared to the control group, rats treated with BeSO_4_ exhibited degenerative changes in the spleen tissues including: a thickening of the marginal area, localized central dilatation of the spleen nodules, red pulp with blood stasis, and the nuclear fragmentation which indicates necrosis. We observed moderate spleen necrosis in rats receiving of EA at the dose of 100 mg/kg. Treatment with EA at the dose of 300 mg/kg dramatically reduced these pathological indices in the spleen tissue.

## Discussion

Chronic exposure to Be promotes ROS formation and cellular oxidative damage in multiple organs. Various drugs are available to treat beryllium-induced diseases, but their uses are limited due to their side effects and the escalating price. To overcome these problems, natural compounds may act as an alternative therapy. Ellagic acid is a naturally occurring plant polyphenol found in a variety of foods, which is effective in impeding oxidative stress (Yüce et al. 2007; Hadrup et al. 2016). The present study is aimed to investigate the antioxidant potential of EA against BeSO_4_-induced oxidative stress in the spleen.

Spleen is susceptible to environmental pollutants that are immune toxic. It has been reported that exposure to a low-dose mixture of twenty-seven environmental chemicals exhibit enlarged spleens in juvenile male rats (Nirala et al. 2009). Consistently, the current study showed that administration of BeSO_4_ at the dose of 12 mg/kg results in increased spleen weight and significant hematotoxicity, suggesting that spleen is a target organ of beryllium sulphate. Haematologic parameters are a sensitive target of chemical exposure. For example, exposure to formaldehyde significantly decreased the blood WBC, lymphocytes and granulocyte counts in BALB/c mice (Wei et al. 2017); exposure to TiO_2_ nanoparticles led to reduced WBC and RBC in ICR mice (Hong et al. 2017). Consistently, the present study showed that exposure to BeSO_4_ elevated the WBC, Neu, Lym, and PLT counts compared to the control group (all *p* < 0.05) (Table 2). We also found that EA (100 and 300 mg/kg) attenuates the effect of the above-mentioned variables, thus reducing hematotoxicity in BeSO_4_-intoxicated rats. Con A and LPS are stimulants of T and B lymphocyte proliferation (Zeng et al. 2015), and play important roles in the immune response. A previous study demonstrated that exposure to boron increased the splenic T and B populations and enhanced the ovalbumin-and LPS-stimulated cultured splenocyte proliferation in BALB/C mice (Wang et al. 2019), and further investigations showed that exposure to C9-13-CPs increased the percentages of the splenic lymphocytes, T cells and NK cells and enhanced the Con A-stimulated cultured splenocyte proliferation (Gao et al. 2014). Similarly, the current study showed that exposure to BeSO_4_ enhanced both Con A and LPS-stimulated proliferation of splenocytes *in vitro* (all *p* < 0.05) (Figure 2), which agreed with the observation that BeSO_4_ elevated the WBC, Neu, Lym, and PLT counts. This may also underlie the mechanism of the BeSO_4_-induced increase in spleen weight. This implies that BeSO_4_ induced both cellular immunity and humoral immunity. Immune homeostasis is pivotal for normal immune function. The abnormal increases in the lymphocyte proliferation and blood immune cells indicated that the exposure to BeSO_4_ caused potential immunomodulatory effects. Researchers have reported that impairs cytotoxic T-Cell function and suppresses humoral immunity in mice (Allen et al. 2003). Our results showed that administration of EA significantly suppressed the BeSO_4_-induced enhancement of Con A and LPS-stimulated proliferation (all *p* < 0.05), suggesting that EA (more potently at dose 300 mg/kg) restricts the extent of splenic injuries by reducing immunomodulatory effects in BeSO_4_-intoxicant rats. These results might be attributed to the antioxidant activity of EA.

Generally, over production of reactive oxygen species (ROS) contributes to generate oxidative stress. Beryllium induces oxidative stress due to the excess production of hydrogen peroxide and superoxide free radicals (Sawyer et al. 2005). Agrawal et al. (2015) reported that excess production of ROS depletes GSH and induces membrane damage on vital organs. Nirala et al. (2009) demonstrated that metal toxicity leads to the production of ROS, which causes peroxidation of membrane lipids and induces a plethora of alterations in the structure and function of cellular membranes. In this study, decreased GSH after BeSO_4_ administration implicates excess production of ROS due to the failure of the antioxidant defense mechanism. EA acts as an antioxidant and possibly could decrease pro-oxidant effect of BeSO_4_ up to some extent. Administration of EA increased the enzymatic and nonenzymatic antioxidant defense mechanisms more effectively, thus prevented ROS as well as maintained the GSH level more towards normal in the spleen, which is also evidenced by evidences of recovered cellular morphology and restoration of splenic tissue to the native state. ROS and free radicals are involved in a variety of pathological events, including cancer.

The antioxidant defense enzymes play an important role in maintaining physiological levels of oxygen and hydrogen peroxide and eliminating peroxides generated from oblivious exposure to xenobiotics and drugs. SOD is one of the major antioxidant enzymes, which catalyses ROS in most cells. In addition, SOD plays an important role in the elimination of ROS and acts as the key component of cellular defense system against oxidative stress. Mishra et al. (2009) reported that SOD converts superoxide (O_2_^−^) to hydrogen peroxide (H_2_O_2_) and is a major defense system for aerobic cells in combating toxic effects of superoxide radical. The chemical activity of SOD depends on various essential trace elements and prosthetic groups for proper molecular organization and enzymatic action. Beryllium reduces the activities of major antioxidant enzymes, including SOD. El-Beshbishy et al. (2012) demonstrated that Be may bind to the enzymatic active site and inhibit mRNA expression of SOD, thus decreasing SOD activities. In line with these studies, our results showed that BeSO_4_ reduced SOD activity. Singh et al. (2000) suggested that using any natural compound with antioxidant properties may help in maintaining health. It was reported that numerous medicinal plants possess protective effect against beryllium-induced toxicity (Agrawal et al. 2013). EA exhibits its antioxidant effects indirectly by increasing the activity of antioxidant enzymes. For example, EA showed protective effects against sodium arsenate–induced hepatorenal toxicity in rats via increasing the activity of SOD and GPx (Mehrzadi et al. 2019). Moreover, EA enhanced the endogenous antioxidant system to protect against methotrexate-induced hepatotoxicity (Poli et al. 1987). Administration of EA restored the activity of SOD in the present study, suggesting that EA treatment could protect the spleen tissue indirectly via increasing the antioxidant enzymes.

TBARS, such as malondialdehyde (MDA), lipid hydroperoxides (LOOH), propanal, hexanal, and 4-hydroxynonenal (4-HNE), are the end-product of lipid peroxidation, which causes structural and functional alterations in the cells (Ding et al. 2014). Among these, MDA is the most mutagenic product of lipid peroxidation (Esterbauer et al. 1990) and the level of MDA is used as an indirect biomarker of oxidative stress in tissues (Priyamvada et al. 2008). A previous study reported that EA attenuates doxorubicin-induced testicular injury in rats by reducing the production of MDA (Routray and Ali 2016). Our results showed that administration of EA significantly decreased MDA level in the spleen tissue of BeSO_4_-intoxicant rats. Decreased levels of MDA indicates the protective effect of EA on lipid peroxidation.

It is well known that the beryllium generates oxidative stress and ultimately leads to the initiation of apoptosis (Kittle et al. 2002). Various natural resources and compounds have been reported for their protective effect against beryllium-induced toxicity (Agrawal et al. 2015). In this study, qRT-PCR and Western blot were performed to demonstrate the effect of EA on the apoptosis induced by BeSO_4_. Expression of apoptotic markers such as Bax, caspase-3, PARP and Bcl-2 was studied. The balance between the expression levels of pro-apoptotic (Bax) and anti-apoptotic protein (Bcl-2) is important for the regulation of cell death and survival mechanisms. Moreover, Thornberry (1997) demonstrated that caspase-3 is involved in the activation of cascade pathways during apoptosis. Our results from qRT-PCR and western blotting experiments demonstrated the protective effect of EA against BeSO_4_-induced apoptosis through a dose dependent increase in Bax and decrease in Bcl-2, inhibition of caspase-3 activation and prevention of PARP activation. Thus, this experiment verified that EA attenuates apoptosis in the spleen via antioxidant.

Morphological architecture of the spleen samples was further confirmed through haematoxylin and eosin staining. The morphology of the spleen samples was observed using haematoxylin eosin staining, which indicated the changes between sections of different groups. Our histopathological observations demonstrated structural changes in the spleen tissue of BeSO_4_-treated rats. EA was found to be effective in curing spleen alteration such as thickening of the marginal area, localized central dilatation of the spleen nodules, lymphocyte necrosis, nuclear fragmentation, and red pulp with blood stasis (Figure 5). These results indicated the beneficial effects of EA on BeSO_4_-induced histopathological alterations in the spleen tissue.

## Conclusions

**Figure 6.**
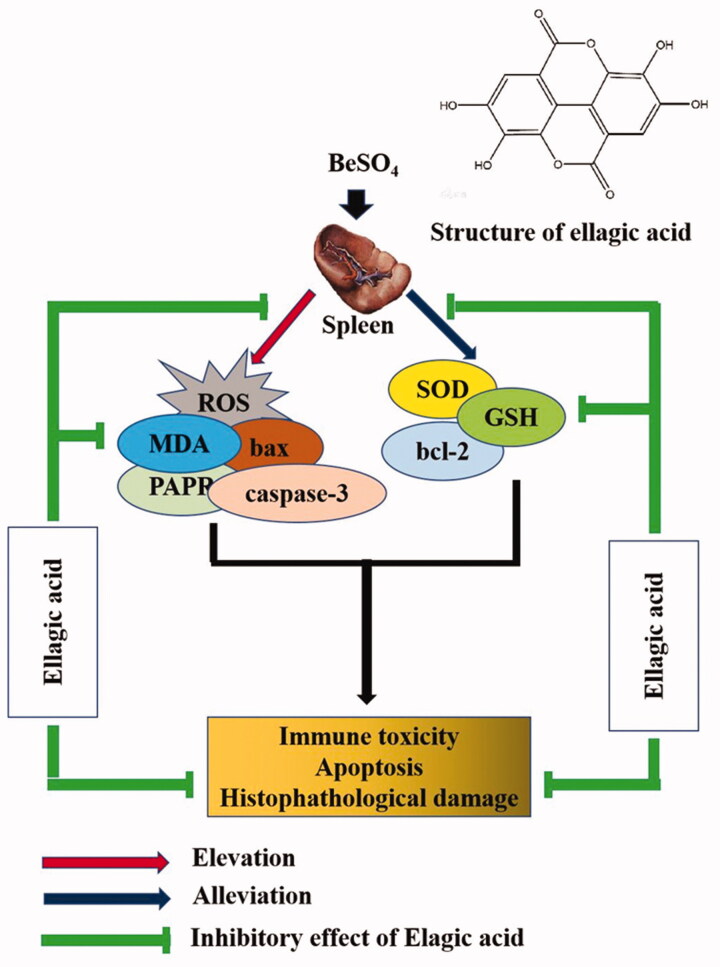
Proposed mechanism of ellagic acid against BeSO_4_-induced splenic toxicity.

The current study demonstrated that EA treatment is effective in alleviating BeSO_4_-induced splenic toxicity. EA could reduce oxidative stress via decreasing the levels of MDA. On the other hand, EA enhances the activity of endogenous antioxidant enzymes such as SOD, GSH and thereby attenuates BeSO_4_-induced splenic toxicity. Collectively, our results suggested that EA provides a safe and natural option for prevention of BeSO_4_-induced toxicity in the spleen. The proposed model indicated the possible ameliorating mechanism of EA against BeSO_4_-induced splenic toxicity (Figure 6). However, further studies are required to precisely understand the underlying molecular mechanisms.
